# Data science approaches to confronting the COVID-19 pandemic: a narrative review

**DOI:** 10.1098/rsta.2021.0127

**Published:** 2022-01-10

**Authors:** Qingpeng Zhang, Jianxi Gao, Joseph T. Wu, Zhidong Cao, Daniel Dajun Zeng

**Affiliations:** ^1^ School of Data Science, City University of Hong Kong, Hong Kong; ^2^ Department of Computer Science, Rensselaer Polytechnic Institute, Troy, NY 12180, USA; ^3^ WHO Collaborating Centre for Infectious Disease Epidemiology and Control, School of Public Health, LKS Faculty of Medicine, The University of Hong Kong, Hong Kong; ^4^ The State Key Laboratory of Management and Control for Complex Systems, Institute of Automation, Chinese Academy of Sciences, Beijing 100190, People’s Republic of China; ^5^ School of Artificial Intelligence, University of Chinese Academy of Sciences, Beijing 100190, People’s Republic of China

**Keywords:** infectious disease, mathematical modelling, data science, big data, COVID-19

## Abstract

During the COVID-19 pandemic, more than ever, data science has become a powerful weapon in combating an infectious disease epidemic and arguably any future infectious disease epidemic. Computer scientists, data scientists, physicists and mathematicians have joined public health professionals and virologists to confront the largest pandemic in the century by capitalizing on the large-scale ‘big data’ generated and harnessed for combating the COVID-19 pandemic. In this paper, we review the newly born data science approaches to confronting COVID-19, including the estimation of epidemiological parameters, digital contact tracing, diagnosis, policy-making, resource allocation, risk assessment, mental health surveillance, social media analytics, drug repurposing and drug development. We compare the new approaches with conventional epidemiological studies, discuss lessons we learned from the COVID-19 pandemic, and highlight opportunities and challenges of data science approaches to confronting future infectious disease epidemics.

This article is part of the theme issue ‘Data science approaches to infectious disease surveillance’.

## 1. Introduction

The use of data science methodologies in medicine and public health has been enabled by the wide availability of big data of human mobility, contact tracing, medical imaging, virology, drug screening, bioinformatics, electronic health records and scientific literature along with the ever-growing computing power [1–4]. With these advances, the huge passion of researchers and practitioners, and the urgent need for data-driven insights, during the ongoing coronavirus disease 2019 (COVID-19) pandemic [5], data science has played a key role in understanding and combating the pandemic more than ever.

COVID-19, caused by the severe acute respiratory syndrome coronavirus 2 (SARS-CoV-2) [6], has swept the globe and claimed over 3.4 million lives as of 19 May 2021. Because of its enormous impact on global health and economies, the COVID-19 pandemic highlights a critical need for timely and accurate data sources that are both individualized and population-wide to inform data-driven insights into disease surveillance and control. Compared with responses to previous epidemics such as SARS, Ebola, HIV and MERS, the COVID-19 pandemic has attracted overwhelming attention from not only medicine and public health professionals but also experts in other data and computational sciences fields that in previous epidemics were more peripheral [7,8].

The COVID-19 pandemic presents a platform as well as a rich data source for mathematicians, physicists and engineers to contribute to disease understanding from data-driven and computational perspectives. Some of these data were unavailable in previous epidemics, while other data were available, but their potential had not been fully unleashed. The public health systems established by many countries’ Centres for Disease Control (CDCs), including those proven to be effective in the past, were easily outflanked by the SARS-CoV-2 virus due to its very high transmissibility and the ever-increasing global human mobility. Within only a few weeks of the virus being reported it was apparent that conventional public health practices had failed in containing it. Looking back, there were notable deficiencies in the public health systems [7,8], including (a) the slow response to highly contagious viruses, particularly if the symptoms resembled those of seasonal influenza and other mild infectious diseases; (b) the lack of reliable data at critical points (such as early outbreak and mutant strains); (c) slow and disorganized data collection; (d) policy decision-making based on political expediency but not scientific evidence; (e) slow and incomplete manual contact tracing; (f) the conflict between the effectiveness of contact tracing and the invasion of privacy; and (g) difficulty in identifying effective drugs to treat COVID-19 patients.

Many of these deficiencies can be addressed by creatively mining big data related to people’s behaviours and opinions, the biological structure of drugs, human interactomes and the constantly mutating virus. The threat of the pandemic has resulted in the whole scientific community being mobilized to combat COVID-19, resulting in many successful and innovative applications. These applications required the capabilities of not only experts in one field but collaborations between people with diverse professional backgrounds. A difficult year has passed, yet it was also a remarkable year of the rise of interdisciplinary data-driven research on emerging infectious diseases. It is therefore important to summarize the progress that has been made so far, and to lay out a blueprint of an emerging field of using data science and advanced computational models to confront future infectious diseases.

In this article, we briefly summarize the important progress made during the COVID-19 pandemic. There have been over 400 000 coronavirus-related publications in 2020 alone [9]. The list of papers we reviewed here (see table 1) is by no means complete, nor is it meant to be. Instead, we selected a set of typical and representative publications and discuss how these approaches shed light on how data science will be an indispensable tool in the ongoing war against the COVID-19 and future epidemics. The selection process is as follows. First, we used the keyword combination (‘COVID-19’ *OR ‘2019-nCov’) *AND (‘data science’ *OR ‘artificial intelligence’) to retrieve all related papers during 1 January 2020 to 31 May 2021 from Web of Science by Clarivate Analytics. Second, we used the same keyword combination to further retrieve additional conference papers from DBLP (a computer sciences bibliographic database). Third, we ranked the retrieved papers in terms of the number of citations and the impact factor of the journals. Fourth, we manually added a small number of papers that we agreed to be representative but not in the highly cited list. Fifth, the authors and five PhD students manually selected the papers to review. We prioritized the representative papers published in top-tier journals.

**Table 1.** Data-driven COVID-19 publications that we reviewed.

section	data	publication
modelling human mobility	human movement data	[10–12]
	migration data	[13]
	nationwide census mobility fluxes	[14]
	open source anonymized human movement data (Baidu migration data)	[15,16]
	aggregated mobile phone users data (provided by SafeGraph)	[17,18]
	anonymized daily mobile phone location data	[19]
	teralytics	[20]
	national census data	[21]
	mobile phone, census and demographic data	[22]
	open government data and Google’s Community Mobility Report	[23]
	digital transactions for transport	[24]
	Google’s Community Mobility Reports	[25]
	near-real-time Italian mobility dataset provided by Facebook	[26]
	air transportation and ground mobility	[27]
	global air travel data	[28]
	mobile phone data	[29]
manual and digital contact tracing	manual contact tracing data in Shenzhen City, China	[30]
	survey data for Wuhan City and Shanghai City and manual contact tracing data in Hunan Province	[31]
	digital contact tracing techniques	[32,33]
	manual and digital contact tracing data	[34]
	online panel survey with mobile tracking data	[35]
empirical evaluation of government responses	Governments’ response	[36–38]
	local/regional/national NPIs data	[39]
	Governments’ response data in Germany	[40]
assessing the economic, trade and supply chain impact	Global Trade Analysis Project (GTAP) dataset	[41–43]
	UN Comtrade dataset	[44]
	World input–output database	[45]
	data provided by the Central Bank of the Republic of Turkey	[46]
	data provided by a major bank in Denmark	[47]
mining patient data	individual-level patient data from official reports in China	[48,49]
	testing data provided by the Israeli Ministry of Health	[50]
	screening data	[51]
	EHR data	[52]
	clinical and laboratory variables	[53]
	chest X-ray images and routine clinical variables	[54]
	computed tomography images	[55]
	potential imaging biomarkers of the CXR radiographs	[56]
	surveys and suicide records	[57]
drug repurposing and development	protein interaction map	[58]
	protein–protein interactions (PPI) dataset	[59]
	experimentally derived PPI data	[60,61]
	databases of drugs, genes, proteins, viruses, diseases, symptoms and their linkages	[62]
	substructure-gene and gene-gene associations	[63]
mining scientific literature	COVID-19 scientific literature dataset	[9,64–70]
	information retrieval test collections TREC-COVID	[71]
	question answering dataset	[67,72]
	questions from FAQ sections of the Center for Disease Control	[68]
	PubMed citation database	[69,73]
Social media analytics and Web mining	information-seeking behaviours	[74]
	Internet searches (Google Trends)	[75]
	Internet searches and social media data	[76]
	social media discussions	[77]
	Google search	[78]
	international survey of risk perception of COVID-19	[79]
	COVID-19 misinformation	[80]

In this article, we first reviewed the publications that used novel data sources/modalities and methods to address a broad spectrum of problems in disease control. Then, we performed bibliographic analysis to highlight the knowledge flow between these publications and the publications cited by/citing them. We conclude the paper with discussions of lessons we have learned so far in leveraging novel data and data science approaches to confront COVID-19 and other emerging infectious diseases.

## 2. Modelling human mobility

SARS-CoV-2 is contagious in humans who are in close contact [6]. There is overwhelming evidence that SARS-Cov-2, similar to other SARS-like coronaviruses, found its way into a human host through an intermediate host in nature. Human contact has then become the main transmission medium [81,82]. As a result, the progression of the epidemic is heavily dependent on human mobility both locally and internationally. This makes the analysis of human mobility data essential to disease surveillance and policy evaluation. Luckily, we now have access to rich human mobility data including population-based census and survey data representing the general travel tendencies of people, as well as individualized mobility data derived from mobile phones, digital transactions and social media.

Reflecting on the early days of the epidemic in Wuhan City, China, the quick outbreak led to severe under-reporting of the problem [83]: on the one hand, many asymptomatic but infected people and people with mild symptoms did not realize that they were infected until they had recovered; on the other hand, many symptomatic people could not be admitted to hospital due to limited healthcare resources. As a result, the early epidemiological data did not fully represent all patients as early reports usually assumed a short serial interval period because they were based on data of severely ill patients who were admitted to hospital, while it missed those who were not hospitalized. It seems that similar situations occurred in other places around the world. As a result, a number of studies used human movement data to estimate the epidemiological parameters, such as the basic reproduction number $R_{0}$, because people travelling out of Wuhan were closely monitored and well described in January and February 2020. [10–12]. Similar migration data were also used to reconstruct the full transmission dynamics of COVID-19 in Wuhan [13].

The success of using human mobility data to estimate the epidemiological parameters of the disease translates to other tasks. Travel restriction has been a popular control measure around the world in response to restricting the spread of SARS-CoV-2. Similarly, Gatto *et al.* used nationwide census mobility fluxes to quantify the effect of local non-pharmaceutical interventions (NPIs) and support the spatio-temporal planning of emergency measures in Italy [14]. However, a number of studies concluded that travel restriction might not be the most effective approach to containing the virus. Lai *et al.* and Kraemer *et al.* used open-source anonymized human movement data (Baidu migration data, https://qianxi.baidu.com/, derived from Baidu users) to evaluate the effect of NPIs in containing the COVID-19 epidemic in China. It found that early detection and timely isolation of infected patients was more effective than travel restrictions and contact reductions [15,16].

A number of companies provide individual or aggregated mobile phone-derived mobility data. In a representative study using aggregated mobile phone users data (provided by *SafeGraph*, https://www.safegraph.com/), Chang *et al.* developed dynamic mobility networks to simulate the COVID-19 outbreak in 10 major metropolitan areas in the USA [17]. Not only did the model predict the superspreader points of interest would account for a majority of the infections but this work also revealed risk inequities that disadvantaged groups suffered, for instance they had a higher risk of infection because they could not reduce their mobility as sharply. Liu *et al.* reported similar findings from a retrospective analysis of the anonymized daily mobile phone location data in China [19]. Two studies using commercial data (*SafeGraph*, Pei *et al.* [18], *Teralytics* https://www.safegraph.com/, Badr *et al.* [20]) reported that social distancing played a central role in mitigating COVID-19 transmission in the USA.

**Figure 1.**
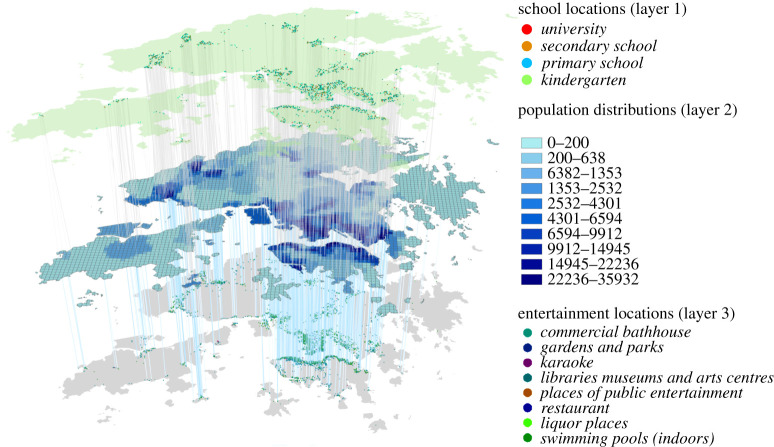
Geographical distribution of the 7.55 million agents and facilities in Hong Kong. Layer 1 represents the distribution of schools. Layer 2 represents the population distribution. Layer 3 represents the locations of entertainment sites. Credit: Zhou *et al.* [23]. (Online version in colour.)

In examining the effect of NPIs in a city or smaller country, agent-based models are useful because of their flexibility and high granularity in modelling travel patterns. To better model the travel tendencies in a city, census and demographic data are required, especially when individualized mobility data are absent. For example, Koo *et al.* used national census data to build an agent-based model of the COVID-19 transmission in Singapore [21]. Similarly, Aleta *et al.* used mobile phone, census and demographic data to build an agent-based model of the COVID-19 transmission in Boston [22]. A recent study took a more aggressive approach, where Zhou *et al.* constructed an agent-based model with 7.55 million agents representing each citizen in Hong Kong [23]. The authors collected open government data including demographics, public facilities and functional buildings, transportation systems and travel patterns (based on census), and also incorporated the real-time human mobility patterns provided by Google’s Community Mobility Report (https://www.google.com/covid19/mobility/). The entire city of Hong Kong was split into 4905 500 m×500 m grids (refer to figure 1 for an illustration). This very detailed model was used to identify the high-value grids for targeted interventions with low disruption of the whole city.

Human mobility data are useful in informing responsive and adjustable NPIs, which can maintain economic productivity. Leung *et al.* used digital transactions for transport to enable real-time and accurate nowcast and forecast of COVID-19 epidemics in Hong Kong [24]. Successful application of such real-time predictions has the potential to maximize economic productivity. Yang *et al.* proposed a simple optimization scheme that considers both the reduction in infections and the social disruption in New York City, and concluded that tight social distancing measures in public places was the key to protect the elderly who are most vulnerable to experiencing severe disease, or death [25]. In a study in Italy, Bonaccorsi *et al.* modelled mobility restrictions as a shock to the economy by harnessing a near-real-time Italian mobility dataset provided by Facebook. These researchers found that mobility contraction was stronger in municipalities with greater inequality and lower income *per capita*, and they subsequently called for fiscal measures that targeted poverty and inequal mitigation [26].

On a global scale, Chinazzi *et al.* proposed a metapopulation disease transmission model that considered both air transportation and ground mobility across 3200 sub-populations in 200 countries and regions. They suggested that early detection, hand washing, self-isolation and household quarantine were more effective than travel restrictions at containing the virus [27]. Gilbert *et al.* used global air travel data to estimate the risk of COVID-19 importation per African country, as well as the preparedness of each country [28].

Facing a global pandemic, coordination between countries/regions is apparently a key in reducing cross-border transmissions. Ruktanonchai *et al.* examined the coordinated relaxation of NPIs across Europe by estimating human movements among European countries by using mobile phone data. They found that coordination of on–off NPIs is indeed important to containing the outbreak across Europe [29].

## 3. Manual and digital contact tracing

Contact tracing is an indispensable method to identify and isolate at-risk people, in an attempt to reduce infections in the community. During the COVID-19 pandemic, most public health practice has still relied on conventional manual contact tracing. Although such data are rarely made publicly available for research due to privacy concerns, there have been good empirical and modelling studies using it. Bi *et al.* analysed a complete dataset of 391 cases and 1286 of their close contacts in Shenzhen City (provided by Shenzhen CDC), China, during 14 January 2020–12 February 2020, and demonstrated that contact tracing significantly reduced the reproduction number and thus prevented a localized outbreak [30]. Zhang *et al.* analysed survey data for Wuhan City and Shanghai City, as well as detailed contact tracing data in Hunan Province (provided by Hunan CDC), and constructed a transmission model to evaluate the impact of NPIs on transmission [31]. They concluded that the NPIs implemented in these places had successfully controlled the COVID-19 outbreak.

Conventional manual contact tracing has major challenges, such as recall bias and time delay. The wide adoption of smartphones makes the novel digital contact tracing techniques a promising supplement to, if not replacement of, manual contact tracing [32,33]. This is particularly relevant to SARS-Cov-2, which is highly infectious. Ferretti *et al.* used a mathematical model to explore the feasibility of controlling the epidemic using conventional manual contact tracing by questionnaires versus digital contact tracing, and concluded that manual contact tracing is not feasible. Thus, the use of digital contact tracing is potentially more effective in stopping the epidemic given the high proportion of people using smartphones [34].

**Figure 2.**
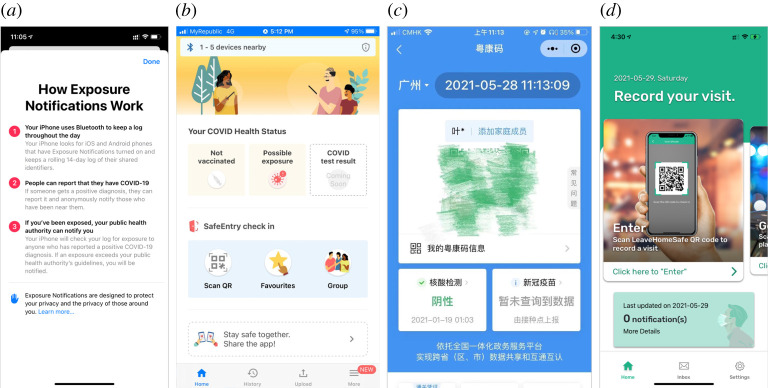
Three typical digital contact tracing apps: (*a*) Apple’s *Exposure Notification* function (Bluetooth-based). (*b*) *TraceTogether* system in Singapore (Bluetooth-based). (*c*) *Health Code* system in Mainland China (Mandatory manual input), (*d*) *LeaveHomeSafe* system in Hong Kong (voluntary manual input). (Online version in colour.)

In developed countries/regions, there appear to be no technical obstacles for effective digital contact tracing because current smartphones are mostly equipped with GPS and Bluetooth [84]. Both Google and Apple have implemented frameworks in smartphones to assist in contact tracing and exposure notifications (figure 2). Since COVID-19 is likely to become endemic, digital contact tracing may eventually become a common public health practice. However, the wide implementation of digital contact tracing has not been particularly successful except for a few countries in East Asia [85]. There are many controversial issues including privacy concerns, accuracy, connection to health authorities, and other cultural and political factors [85,86]. In many lower- and middle-income countries/regions, where citizens are less technologically savvy, manual contact tracing is still playing the dominant role in containing the epidemic.

Since late 2020, Singapore has mandated the use of a digital contact tracing app, *TraceTogether*. In mainland China, different cities/provinces have produced their own *Health Code* systems and these isolated systems are now merging into a nationwide Health Code system. In Hong Kong, a conservative contact tracing app, *LeaveHomeSafe*, has been made available by the government. LeaveHomeSafe does not have access to users’ private data. There is no registration requirement, and it only sends users (not public health authorities) exposure notifications. Its use is voluntary and people can always choose to manually leave their contact information (usually nobody verifies the information) when entering premises (such as a restaurant) that requires it (figure 2). Given Ferretti *et al.*’s simulation research [34], the efficacy of such a voluntary-based digital contact tracing system in reducing transmission is limited by the low proportion of trustworthy data.

How to motivate people to use digital contact tracing is an important public health challenge. Munzert *et al.* combined an online panel survey and mobile tracking data to measure usage of the official contact tracing app in Germany, and found that people with different demographic backgrounds exhibited different usage of the app [35]. These researchers also showed that video messages were not effective in motivating updates, while small monetary incentives may strongly increase updates.

Even if vaccines become widely available, their development may not keep pace with virus mutations. Thus, contact tracing remains a critical tool in stopping the epidemic. To unleash the potential of digital technology to improve contact tracing accuracy, advances are required in both technology and public health research. On the one hand, more advanced technologies are needed to dispel people’s doubts about data privacy, while on the other hand, how to motivate and incentivize people to adopt new technologies (including other interventions and vaccinations) might be the most important question.

## 4. Empirical evaluation of government responses

Governments and authorities around the world responded to the COVID-19 pandemic with a range of NPIs. Compliance with policy measures provide a rich dataset of lessons and experiences that are in valuable for future decision-making. A number of studies have quantified the extent of the action, as well as the compliance with policy measures. A typical example is *Oxford Covid-19 Government Response Tracker* (*OxCGRT*, https://www.bsg.ox.ac.uk/research/research-projects/covid-19-government-response-tracker), which collects systematic information on more than 180 countries’ policy measures since 1 January 2020. More specifically, OxCGRT records these policies on a scale to reflect the extent of government action, and policy indices are created based on the scores [38]. Similarly, Porcher published *Response2covid19* (https://response2covid19.org/), a dataset of governments’ response to the COVID-19 pandemic [36]. Another global dataset, the *Citizenship, Migration and Mobility in a Pandemic* (*CMMP*, https://www.cmm-pandemic.com/) was introduced by Piccoli *et al.* [37]. Quantifying the effect of various NPIs is another important problem. Hsiang *et al.* compiled data on 1700 local/regional/national NPIs deployed in six countries, and applied reduced-form econometric methods to empirically measure the effect of these NPIs on flattening the epidemic curve [39]. Dehning *et al.* analysed the data in Germany using a Bayesian inference model and emphasized that relaxation of NPIs should be undertaken warily, because the currently deployed NPIs had barely contained the outbreak [40]. However, there is little research that compared the implementation and uptake of NPIs across different countries. Objective and data-driven evaluation of the actual NPIs deployed around the world is crucial for decision-makers to confront future infectious disease epidemics. Moreover, with the growing accessibility to vaccines, another important question arises: how to effectively and efficiently allocate vaccines locally and globally. This question has not been well addressed by the time of this review, and the authors would like to call for data-driven research on this crucial topic.

## 5. Assessing the economic, trade and supply chain impact

Travel restrictions and NPIs have dramatically affected the global supply chains and trades. Guan *et al.* adopted the latest economic disaster modelling to examine the supply chain effects of a set of NPIs scenarios. They found that the supply chain losses were dependent on the number of countries imposing travel restrictions, while a longer containment that might control the epidemic could impose smaller losses [41]. This study built the global supply chain network using the *Global Trade Analysis Project (GTAP)* database [42], which is subject to a subscription fee. Maliszewska *et al.* also used GTAP data and previous episodes of global epidemics to simulate the impact of the COVID-19 pandemic on gross domestic product and trade, and drew similar conclusions [43]. More recently, Ye *et al.* developed an integrated network model to investigate the personal protective equipment (PPE) shortage contagion patterns on a global trade network harvested from the World Customs Organization report, and found that PPE export restrictions exacerbated shortages, and caused shortage contagion travelling faster than disease contagion [44]. Malliet *et al.* used a computable general equilibrium model to assess the impacts of French NPIs on environmental and energy policies at macroeconomic and sectoral levels, and found that lockdown measure decreased economic output but generated positive environmental impact by reducing CO_2_ emissions [45]. In other two studies, Çakmaklı *et al.* and Andersen *et al.* quantified the macroeconomic effects of COVID-19 on consumers and economies by harnessing the data provided by the Central Bank of the Republic of Turkey [46] and a major bank in Denmark [47], respectively.

## 6. Mining patient data and drug repurposing

Mining patient data can generate enormous amounts of valuable information, ranging from aggregated statistics on a daily or weekly basis to detailed electronic health records (EHRs). Analysing the time series of case counts has always been the focus of epidemic modelling. Xu *et al.* collected and curated individual-level patient data from official reports in China, and published it for public use [48]. This dataset has successfully enabled a dozen of downstream epidemiological studies. In another study, Bednarski *et al.* explored how to use reinforcement learning and deep learning models to derive the near-optimal redistribution of medical equipment to support public health emergencies [49].

How to prioritize testing for COVID-19 is important because testing resources are usually limited. To this end, Zoabi *et al.* developed a machine learning model to predict the COVID-19 diagnosis based on the testing data provided by the Israeli Ministry of Health [50]. In another study, Callahan *et al.* used screening data to address the same problem by developing a machine learning model [51]. In dealing with the patients admitted to the hospital, the major challenge is to prioritize the patients with severe disease and a high risk of death. The ability to derive an accurate individual-level risk score on the EHR is crucial for effective resource allocation and distribution, and prioritizing vaccination programs. Estiri *et al.* trained age-stratified generalized linear models with component-wise gradient boosting to predict the death of patients before getting infected [52]. In a population-based study from Hong Kong, Zhou *et al.* developed a simple risk score for predicting severe COVID-19 disease using clinical and laboratory variables [53].

Machine learning has been recognized as effective in predicting the risk of a range of patient outcomes. It is particularly useful for COVID-19 because the diagnosis usually involves both structured data and medical imaging data. Shamout *et al.* developed deep neural network models to predict deterioration risk by learning from chest X-ray images and routine clinical variables [54]. Wang *et al.* proposed a deep learning-based AI system for COVID-19 diagnostic and prognostic analysis by analysing computed tomography images, and validated the model on a Chinese dataset of 5372 patients [55]. Oh *et al.* proposed a patch-based convolutional neural network method for COVID-19 diagnosis by analysing the potential imaging biomarkers of the CXR radiographs [56]. The success of using deep learning and more general machine learning techniques in COVID-19 diagnosis and prognosis, and patient stratification continues. Please refer to the latest review of these techniques [87].

Owing to people’s isolation during the COVID-19 pandemic, mental health has emerged as another focal issue [88–90]. Surveys and suicide records could provide a good data source if they were collected during the time period of the pandemic. For example, Holman *et al.* examined mental health issues during the COVID-19 pandemic by sampling US citizens across three 10-day periods, and identified a number of factors associated with acute stress and depressive symptoms [57]. However, due to the difficulty in obtaining reliable data, data science and machine learning approaches that accurately detect mental health issues during the ongoing COVID-19 pandemic remain under-researched. There are a few successful studies, which are mostly based on Internet and social media data, rather than individual patients’ records.

Because of the speed of onset, and size of impact of COVID-19, repurposing currently is an efficient way of ensuring that effective treatment is available . Early in the pandemic, Gordon *et al.* showed that a protein interaction map of SARS-CoV-2 could identify targets for drug repurposing [58]. In the search for drug candidates in the sea of biological data, with a focus on protein–protein interactions (PPIs), network science and machine learning have the advantage of being able to model the high-dimensional biological and pharmaceutical data associated with different drugs. Sadegh *et al.* developed an online interactive platform named *CoVex* (https://exbio.wzw.tum.de/covex/) for COVID-19 drug or target identification by integrating virus–human protein interactions, human PPI, and drug-target interactions [59].

**Figure 3.**
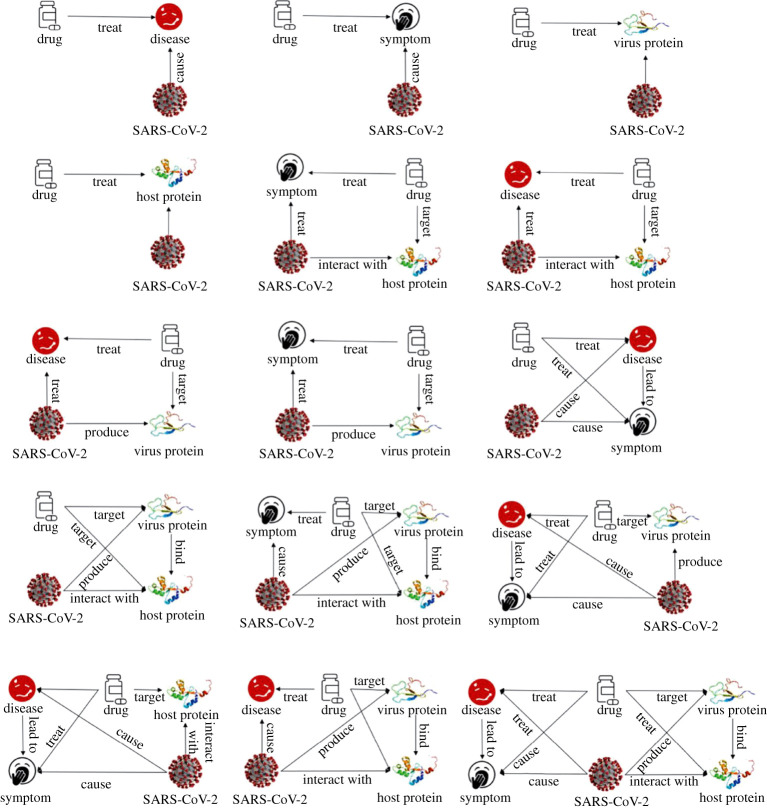
Motifs-of-interest for drug repurposing in a knowledge graph: a knowledge graph is a multi-relational graph composed of entities and relations. Each entity represents a specific protein, gene, drug, virus, disease or symptom and each relation represents a known existing linkage between any two entities. A motif is a connected subgraph representing fundamental building block of the knowledge graphs. Motifs-of-interest are defined based on their importance to the drug repurposing task. Motif-clique discovery algorithms are used to extract these defined motifs-of-interest. Credit: Yan *et al.*/Wiley [62]. (Online version in colour.)

In a representative study, Gysi *et al.* adopted a set of machine learning, network diffusion, and network proximity models to prioritize 6340 drugs that might treat COVID-19 [60]. These authors constructed the human interactome with 18 505 proteins and 327 924 protein interactions by harvesting 21 public databases that compile experimentally derived PPI data. The authors found that no single model consistently outperformed others across all datasets, and thus a multimodal approach was used to perform model fusion for the best prediction performance. A similar study was carried out by Zhou *et al.* [61], where high-value proteins and drug combinations were derived by a network-based algorithm. Yan *et al.* proposed a knowledge graph approach to prioritise drug candidates against SARS-Cov-2 [62]. This study integrated 14 biological databases of drugs, genes, proteins, viruses, diseases, symptoms and their linkages, and developed a network-based algorithm to extract hidden linkages connecting drugs and COVID-19 from the constructed knowledge graph. See figure 3 for the description of the knowledge graph and the identified motifs-of-interest. Pham *et al.* proposed a deep learning method, namely *DeepCE*, to model substructure–gene and gene–gene associations for predicting the differential gene expression profile perturbed by de novo chemicals, and demonstrated that DeepCE outperformed state-of-the-art, and could be applied to COVID-19 drug repurposing of COVID-19 with clinical evidence [63]. Zhou *et al.* provided a useful review and helpful illustrations of these machine learning, and AI techniques for COVID-19 drug repurposing [91] The knowledge graph does not have to be manually constructed, except for the existing biological datasets, as machine learning and natural language processing (NLP) techniques are appropriate tools to automatically construct knowledge graphs from scientific literature [65].

## 7. Mining scientific literature

The COVID-19 pandemic has led to a huge corpus of coronavirus-related publications across disciplines. There were over 400 000 publications about COVID-19 and SARS-Cov-2 in 2020, and the number is ever-growing. Mining this huge set of scientific articles can facilitate knowledge discovery, enable novel expert systems, identify research trends and guide research policy.

There are a number of open-source datasets of COVID-19 scientific literature. *TREC-COVID* (https://ir.nist.gov/covidSubmit/) is a set of information retrieval test collections jointly organized by the Allen Institute for Artificial Intelligence (AI2), the National Institute of Standards and Technology, the National Library of Medicine (NLM), Oregon Health & Science University, and the University of Texas Health Science Center at Houston [71]. TREC-COVID provides a list of papers contributed by the challengers (https://ir.nist.gov/covidSubmit/bib.html), but the list seems incomplete. AI2, in collaboration with Chan Zuckerberg Initiative, Georgetown University, Microsoft, IBM, NLM, and the White House of the USA, also created the *COVID-19 Open Research Dataset Challenge* (*CORD-19*, https://www.kaggle.com/allen-institute-for-ai/CORD-19-research-challenge) through Kaggle [64]. Note that there are over 30 000 COVID-19-related data challenges in Kaggle as of 15 May 2021 (https://www.kaggle.com/search?q=covid-19). MIT Operations Research Center is also maintaining a service, namely the *COVID Analytics* (https://www.covidanalytics.io), which provides a dataset of COVID-19-related papers, with a visualization tool for users to derive their own insights from the data. COVID Analytics has great impact on not only disease surveillance, but also the vaccine development. Developers of the *Johnson & Johnson* COVID-19 vaccine and the MIT researchers applied machine learning to help guide the company’s research efforts into a potential vaccine by analysing COVID Analytics data and other real-world data. For example, they worked together to identify key locations to set up trial sites for the company (https://news.mit.edu/2021/behind-covid-19-vaccine-development-0518).

**Figure 4.**
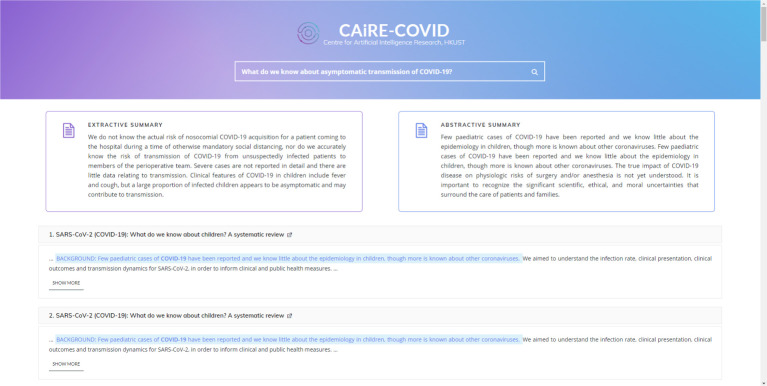
An example of the answers and summary provided by CAiRE-COVID. Screenshot taken by searching ‘What do we know about asymptomatic transmission of COVID-19?’ on CAiRE-COVID [72]. (Online version in colour.)

Esteva *et al.* created a semantic search engine, *CO-Search* (http://einstein.ai/covid), which is able to handle complex queries over the COVID-19-related literature [9]. CO-Search has a multi-stage framework, with a hybrid semantic–keyword retriever based on the popular *BERT* language model, and a re-ranker that further sort the order of retrieved documents by relevance. The authors demonstrated the strong performance of CO-Search on the TREC-COVID dataset. Su *et al.* developed a real-time question answering (QA) and document summarization system, namely *CAiRE-COVID* (https://demo.caire.ust.hk/covid/) [72], which is able to answer high-priority questions with question-related information (see figure 4 for an example). Similar to CAiRE-COVID, there are a number of COVID-19 specific QA systems [66–68], and search engines [70]. Machine learning and NLP methods to construct knowledge graphs by analysing the coronavirus-related literature. More specifically, Chen *et al.* combined the CORD-19 dataset [64] and the PubMed dataset [73] to identify COVID-19-related experts and bio-entities [69]. Another example is the *COVID-KG* framework, which could extract fine-grained multimedia knowledge elements from scientific literature [65]. The resulted knowledge is available at http://blender.cs.illinois.edu/covid19/.

## 8. Social media analytics and Web mining

The World Wide Web and social media have become important channels for laymen to retrieve health-related information. There is strong evidence that users’ online behaviours are associated with their health conditions and thus could be used to estimate the epidemic of infectious diseases [92,93]. It is possible that the Web and social media data could inform more timely responses since traditional manual reporting systems have significant lag times. In an empirical study, Bento *et al.* examined people’s information-seeking behaviours in response to the first confirmed COVID-19 case in each state of USA, and found that searches for certain terms were strongly influenced by the timing of the first confirmed case in a state [74]. In a correlation analysis, Effenberger *et al.* found that Internet searches (Google Trends) are correlated with the number of COVID-19 cases across European countries [75]. There was usually a time lag of 11.5 days, indicating that the Internet searches were possibly predictive of actual cases within that time period in Europe. Li *et al.* performed a comprehensive study using both Internet searches and social media data to predict the COVID-19 incidence in China [76]. The authors used both Google Trends and Baidu Index to characterize the popularity of COVID-19-related terms in Internet searches, and the Sina Weibo Index to characterize that in social media interest. The results showed that all three sets of data were correlated with the actual COVID-19 cases in China. Of note however was that the Baidu Index and Sina Weibo Index could predict the outbreak over a week earlier, possibly because Google is not a mainstream search engine in China.

In addition to disease surveillance, the Web and social media have also become a battlefield of truth, rumours, misinformation and even disinformation [80]. Li *et al.* analysed the social media discussions on Sina Weibo and found that specific linguistic and social network features could predict the reposted amount of different types of information [77]. However, the ever-present question was whether the online information was of good quality? To answer this question, early on in the outbreak (as of 6 February 2020), Cuan-Baltazar *et al.* manually screened the COVID-19-related websites by searching relevant terms on Google, and found that the quality and readability of retrieved information was mostly poor, highlighting the risk of the Internet as a public source of information on health [78]. Roozenbeek *et al.* examined predictors of misinformed belief about COVID-19 and SARS-Cov-2, using a dataset of samples from the United Kingdom, Ireland, the USA, Spain and Mexico, identifying a consistently high proportion of misinformed public belief views in all five countries [79]. Such susceptibility to misinformation was found to make people less likely to comply with NPIs or to seek COVID-19 vaccines, suggesting interventions are required to help the public gain trust in science.

Ye *et al.* built a mathematical model, which indicates that the media and opinion leaders should provide true and quality information to the public so that people are willing to comply with public health guidance to protect themselves and the whole population [94]. To achieve this, more rigorous research on mis- and disinformation about COVID-19 is much-needed, especially while facing the rise of populism and anti-scientism worldwide [95,96].

## 9. Discussion

**Figure 5.**
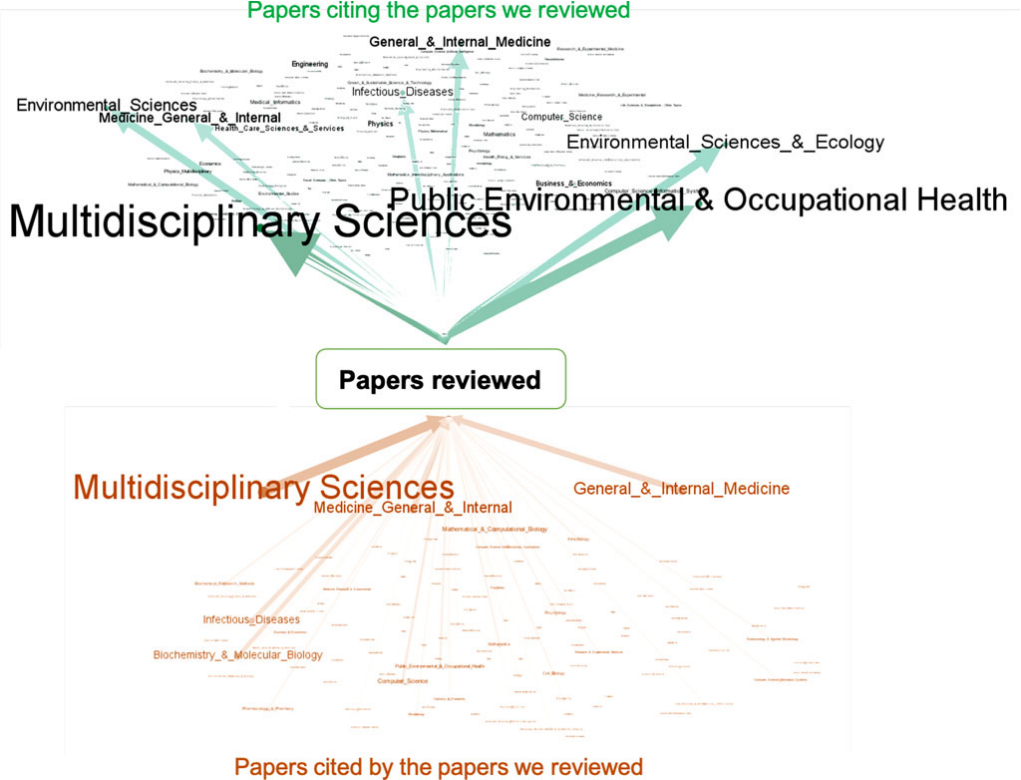
Knowledge transfer from the disciplines of the papers cited by the papers we reviewed (down) to the disciplines of papers citing the papers we reviewed (up). The size of arrows represents the frequency. (Online version in colour.)

**Figure 6.**
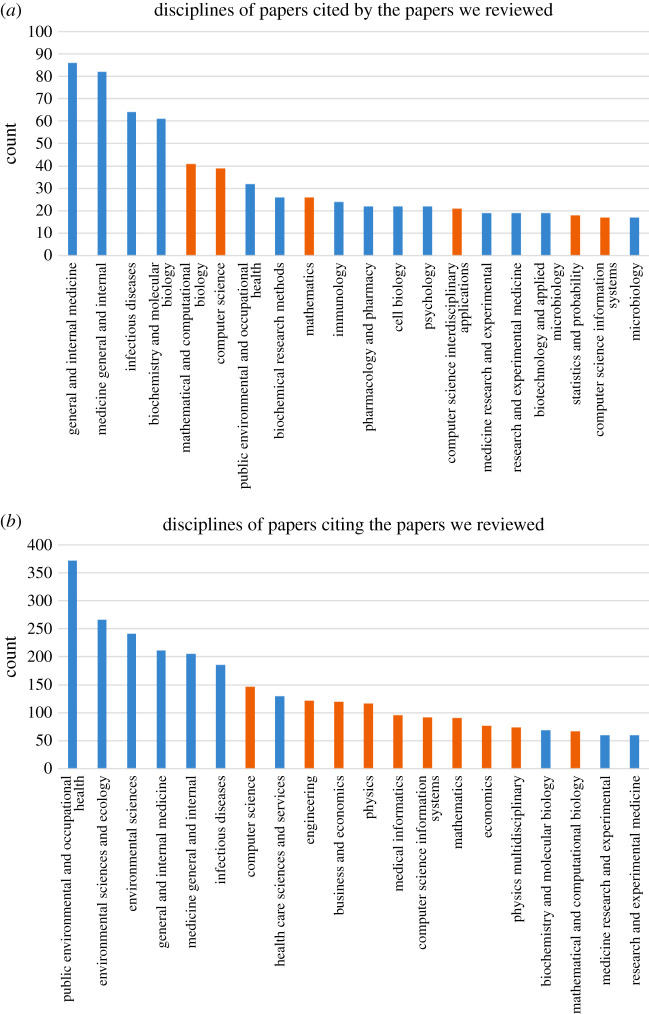
The count of top 20 disciplines (excluding *Multidisciplinary Sciences*) of (*a*) the papers cited by the papers we reviewed, and (*b*) the papers citing the papers we reviewed. The orange bars represent disciplines other than medicine, biology and public health disciplines. (Online version in colour.)

We performed a bibliographic analysis of the papers reviewed above. Figure 5 visualizes the knowledge transfer from the disciplines of the papers cited by the papers we reviewed (*cited-papers*) to the disciplines of papers citing the papers we reviewed (*citing-papers*). The disciplines were determined by the Web of Science (WoS) and one paper may have multiple disciplines. The cited- and citing-papers were also retrieved from WoS. It is obvious that *Multidisciplinary Sciences* is the dominating discipline for both groups of papers. To have a better understanding, we further present the bar charts of these papers’ disciplines excluding *Multidisciplinary Sciences* in figure 6. We found that 6 out of 20 most frequent disciplines of the cited papers were not in medicine, biology or public health. For citing papers, half were not in medicine, biology or public health. Most of these fields are computational sciences. These bibliographic analysis results suggest that COVID-19 research is highly multidisciplinary and there is strong evidence of knowledge transfer between different disciplines.

The impact of the COVID-19 pandemic on human society and scientific community is unprecedented. To win the war against the COVID-19 pandemic requires innovative collaborations between scientists from many disciplines. Data scientists have already shown that by joining with medicine and public health scholars they can identify, analyse and model traditional and novel data generated by, or associated with, the pandemic to produce rich understandings. The innovative use of these data has led to many important applications, that cannot be adequately covered by a single article. In this paper, we selected a set of publications that represent the data science studies in modelling human mobility, developing digital contact tracing techniques, evaluating government responses, assessing the economic impact, mining patient data, drug repurposing, mining scientific literature, social media analytics and Web mining. There are a number of topics that are not covered in detail because of insufficient publications, such as vaccine prioritization [97,98] and vaccine hesitancy [99], screening chatbot [100], crowdsourcing and the emerging folk science. As the pandemic, and research into it, progresses, more knowledge will become available in these topics.

This rich literature of data science approaches to combating the COVID-19 pandemic has provided valuable knowledge, experience and more importantly toolkits that we may use to improve disease surveillance and refine NPIs for COVID-19. The excitement that lies ahead for scientists in all disciplines is the use of these approaches to prevent the outbreak of future infectious diseases. The capability will not only depend on the methodological advances in AI and machine learning, but also on the identification of more data, the linkage across datasets, and the balance between individual’s privacy and the population’s well-being. Research policy-makers should recognize the urgent need for multidisciplinary COVID-19 research and foster novel collaborative research by thematic prioritization of funding and organizing work groups and conferences of researchers from different domains. It is important that the public’s trust in science is secured, so that when the world faces another emerging infectious disease in the future, reactions will be timely, effective and underpinned by believable data-driven NPIs, with which people comply because of their credibility.

## Data accessibility

This article has no additional data.
